# The economic costs of limited health literacy in China: evidence from China’s National Health Literacy Surveillance data

**DOI:** 10.1186/s12913-022-07795-9

**Published:** 2022-04-20

**Authors:** Lefan Liu, Jing Huang, Guoxing Li, Zhuo Chen, Tianfeng He

**Affiliations:** 1 Centre for Health Economics, School of Economics, University of Nottingham Ningbo China, Ningbo, 315100 China; 2 Department of Occupational and Environmental Health Sciences, School of Public Health, Peking University, 38 Xueyuan Road, Beijing, 100191 China; 3 Collge of Public Health, University of Georgia, Athens, GA 30606 USA; 4 Ningbo Municipal Center for Disease Control and Prevention, Ningbo, 315010 China

**Keywords:** Limited health literacy, Out-of-pocket, Medical costs, Health expenditure

## Abstract

**Background:**

Limited health literacy is a public health challenge contributing to the rising health care costs. We assess the economic costs of limited health literacy in China using data from the National Health Literacy Surveillance survey.

**Methods:**

Our data includes a sample of 6316 residents aged 15–69 years old living in Ningbo, China, in 2019. We use box plots to examine the distribution of out-of-pocket health expenditure by the level of health literacy. We then use the estimates from a two-part model to assess the contribution of limited health literacy to individual medical spending and the aggregate health expenditure at different levels of health literacy for the adult population in Ningbo.

**Results:**

Medical costs of limited health literacy are about 10% (177 CNY or about 25 USD) of the annual medical expense of a resident aged 15–69 living in Ningbo. The medical cost of limited health literacy is greater among the rural, female, and older groups than others. If the proportion of people with adequate health literacy increases from 22 to 30% (the target level by 2030), the aggregate out-of-pocket health expenditure in Ningbo will decrease by 100 million CNY (14 million USD), or 0.88% of the 2019 Ningbo government expenditure on health care.

**Conclusions:**

This paper highlights the direct and indirect economic costs associated with limited health literacy. The results should help policymakers evaluate the cost-effectiveness of relevant programs that aim to improve residents’ health literacy.

**Supplementary Information:**

The online version contains supplementary material available at 10.1186/s12913-022-07795-9.

## Background

Health literacy (HL) is a topic of growing importance in the field of public health. Health literacy is defined as an individual’s ability to understand and act on health-related information, or more formally by the US National Library of Medicine, “the degree to which individuals can obtain, process, and understand the basic health information and services they need to make appropriate health-related decisions” [1]. Following this definition, lower health literacy is often associated with poorer health outcomes, and there is growing literature on this topic, although evidence on the causal pathway is often mixed [2]. Many of these studies find limited health literacy is associated with poor health knowledge, increased incidence of chronic illness, poor intermediate disease markers, and insufficient use of preventive health services [3–6]. These studies help us understand one category of the costs associated with limited health literacy: individual burden of diseases. Fewer studies, however, have addressed another category of costs, i.e., medical costs. Medical costs represent the financial burdens to individuals, society, and the health care system. In addition to medical costs, limited health literacy might also be associated with indirect costs such as productivity loss. For example, adults with poor health might have more missed days of work/study due to illness. In general, there are myriad of costs associated with limited health literacy in the form of personal and medical costs. As a result, promoting health literacy is a public health goal in many countries, and interventions to improve health literacy are often prioritized [7, 8]. This is particularly true for China [8].

The first “National Health Literacy Surveillance” (NHLS) survey was conducted in 2008 as part of the ongoing national health literacy promotion effort with annual funding of at least 40 million USD [8]. The first NHLS surveyed around 80,000 residents aged 15–69 in 31 provinces (or equivalents). The proportion of persons with the health literacy level defined as “adequate” is merely 6.48% in 2008, meaning less than 7 out of 100 residents aged 15–69 have adequate health literacy skills. This health literacy rate is not necessarily comparable with indicators in other countries as the instruments and threshold used in classifying adequate levels are different.^1^ However, a large rural-urban disparity exists, with the rate of health literacy being 9.49% among urban residents and only 3.43% among rural residents [11]. The second NHLS survey was conducted in 2012 (surveying 102,985 residents aged 15–69), and the survey has run annually since then. This rate of health literacy rose steadily from 8.8% in 2012 to 10.25% in 2015. In 2016, the Chinese government issued its “Healthy China 2030 Framework”, which proposed major health indicators to be achieved by 2030. In this framework, the national health literacy rate is aimed to reach 30%, tripling the existing level in 2015. Our primary goal is to estimate the medical costs associated with limited health literacy using NHLS survey data.

It is worth noting studies of medical costs associated with limited health literacy are more common in high-income countries than in low-and-middle-income countries (LMICs). According to WHO Health Expenditure Global Report 2020, the 2018 health spending per capita is $3313 in high-income countries but ranges from $40–$466 in LMICs [12]. In LMICs, however, the social security provision is often inadequate, and the share of out-of-pocket health expenditure is high. In 2018, the out-of-pocket share was on average 41–42% in low-income and lower-middle-income countries, 35% in upper-middle-income countries, and 21% in high-income countries [12].^2^ In addition, many of the studies used patient-level data (see [14, 15], for example), which provide a conditional estimate of the health expenditure associated with limited health literacy for a patient population (often with a small sample of patients who received medical treatment). Though the medical expenditures are accurate, such samples provide a limited representation of the general population. Our data are nationally representative and include detailed information on residents’ health literacy regardless of their disease conditions. In the survey, the respondents were asked about the annual out-of-pocket health expenditure, which enables us to examine the extent to which limited health literacy is associated with increased out-of-pocket health expenditure. Therefore, our study will shed light on the potential benefit of health literacy intervention programes that aim to improve residents’ health literacy.

## Methods

### Data and sample selection

Our data comes from the 2019 National Health Literacy Surveillance (NHLS) survey conducted in Ningbo, the second-largest city in Zhejiang province. The 2018 GDP per capita for Zhejiang province ranked fifth among 31 provinces in China [16]. Ningbo is also an important commercial and financial hub in South China [17]. Located in the Yangtze River Delta in South China, Ningbo was ranked as the world’s fourth-largest port city in 2013 [18]. It is only 230 km south of Shanghai (China’s largest city) and well-connected by flight and high-speed train with other major cities in China, such as Beijing (1360 km) and Guangzhou (1350 km).^3^ The total population in Ningbo is 8.54 million at the end of 2019 (the population with a household registration is 6.08 million) [19]. Our sample is representative of the residents aged 15–69 years old who had lived in Ningbo for more than 6 of the previous 12 months, regardless of whether they had local household registration. The age range of 15–69 years old was adopted by the National Institute of Health Education, which was responsible for survey design.

We used a stratified multi-stage PPS (probabilities proportional to population size) sampling frame. There are ten districts (or counties) in Ningbo. At each county, we selected four communities (or townships), and then selected two subdivisions (or villages) within each community (or township) based on PPS. All subdivisions within each community represent urban regions, and all villages within each township represent rural regions. If there were greater than 750 but less than 1500 households within a subdivision (or village), the unit was regarded as a primary sampling unit (PSU). If a selected subdivision (or village) had more than 1500 households, it was divided into several units, each with roughly 750 households. One of the units was randomly selected and used as a PSU. Our surveyors constructed a list of households by field trips in each PSU, from which 120 households were randomly selected. One resident household member aged 15–69 was selected randomly in each household. In each household, all eligible (15–69 years) household members (i.e., who had been living there for more than 6 of the previous 12 months) were grouped by gender and age. One member was then selected for the survey by a Kish grid [20]. A total of 6454 respondents were interviewed, covering 124 subdivisions/villages and 45 communities/townships across ten districts/counties in Ningbo. In the 2019 survey conducted in Ningbo, we incorporated a series of questions on individual out-of-pocket health spending. Our final sample includes 6316 respondents (aged 15–69) surveyed in 2019, after dropping sequentially those with non-response to out-of-pocket health expenditure information (*n* = 74), non-response to health outcome variables (*n* = 29), and non-response to demographic and socioeconomic status information (*n* = 35).

### Measures

#### Questionnaire design and health literacy

The questionnaire was developed based on “Basic Knowledge and Skills of People’s Health Literacy (pilot edition),” which is also called “66 Tips of Health: Chinese Resident Health Literacy Manual” [21]. The 66 Tips of Health document was designed by experts in public health, health education and promotion, and clinical medicine using the Delphi method [22]. Based on the 66 Tips of Health document, a standardized question bank was constructed. The final questionnaire was compiled by the National Institute of Health Education, National Health Commission of China. Compared with the 2008 initial survey, the 2012 survey improved the structure and content of the questionnaire (e.g., decreased the total number of instruments from 96 items to 80 items and expanded scope to assess residents’ ability to obtain medical and health information, process media health information, understand drug specifications, and understand medical science articles) [23]. The questionnaire has kept a similar format since 2012. It is worth noting, to ensure comparability, questions were selected from a question bank to make surveys comparable across survey years [20]. The National Institute of Health Education has approved the reliability and validity of the health literacy scale. The instruments used in the 2012 questionnaire have Cronbach’s alpha of 0.931 and Spearman-Brown split-half coefficient 0.808 [20].^4^

There are 50 health literacy questions in the 2019 national questionnaire, divided into three types: true-or-false; multiple-choice questions (with either one or multiple correct answer(s)); and vignette questions. The correct response to each true-or-false and single-answer question receives one point, and the correct response to a multiple-answer question receives two points towards the total score, with the maximum possible score at 66. Questions can be divided into six categories by their relevance to public health: (1) scientific views of health; (2) infectious disease prevention; (3) chronic disease prevention; (4) safety and first aid; (5) medical care; and (6) health information. We provide examples of the health literacy questions in Table A1 in the Supplementary file (readers can also refer to Appendix B in the Supplementary file for the English version of the questionnaire). A respondent is defined as having adequate health literacy if the respondent obtained at least 80% of the full score (i.e., 53). We choose this threshold (80% of the full score) because it has been used consistently over time since the first national survey by the National Institute of Health Education, making it possible to compare our study with other studies that used the same survey. By design, these questions are grouped into one of the three dimensions: (1) knowledge and attitude (22 items); (2) behaviour and lifestyle (16 items); and (3) health-related skills (12 items). Following the 80% threshold rule, we can define whether a respondent has adequate health literacy on each of the three dimensions.

#### Demographic and socioeconomic status variables

Control variables include variables that represent the demographic characteristics and social-economic status information of the respondents, including the rural/urban classification of residence, age, gender, marital status, number of household members, annual household income per capita, education level, and job status. Age is categorized into three groups as 15–44, 45–59, and 60–69. Annual household income per capita is annual household income divided by household size. Education level is categorized into three levels: low (finished primary school or lower), middle (middle/high school), and high (college or higher). Job status is categorized into five groups: working in public sectors (including those whose responses are civil servants, medical workers, teachers, other state enterprises, or students), farmers, manual laborers, working in private sectors, and the other (including those who are unemployed, retired or not working).

#### Measures of health outcomes

We measure the health and health behaviour of an individual using the following variables:

1. Chronic disease incidence. Respondents were asked whether they had any chronic disease and the name of the disease from a list of six categories, including hypertension, heart problems, cerebrovascular diseases, diabetes, malignant tumour (cancer), and others. The five specific types of diseases were common in Ningbo, and cardiovascular diseases, cancer, and diabetes are the leading cause of death worldwide [25, 26]. The original question asked in the survey is presented in Table A2 in the Supplementary file.
2. Self-reported health status. Respondents were asked to rate their health in general as being: (1) ‘excellent’, (2) ‘good’, (3) ‘fair’, (4) ‘poor’, or (5) ‘very poor’. This five-point Likert scale measure is a robust predictor of mortality and correlates strongly with other objective health indicators [27–29]. We also create a dichotomous measure, which is coded 1 if the response was good/excellent and 0 if the response was fair/poor/very poor.
3. BMI. Respondents were asked about body weight and height, allowing us to compute their Body Mass Index (BMI) (defined as weight in kilograms divided by height in meters squared). In line with the Working Group on Obesity in China [30], we group our respondents into one of the four groups according to their score in BMI: (1) underweight if *BMI <* 18.5, (2) normal if 18.5 ≤ *BMI <* 24; (3) overweight if 24 ≤ *BMI <* 28; and (4) obese if *BMI* ≥ 28.
4. Health behaviour. The survey collected information on two types of health behaviour: whether the respondent is a current smoker; and whether the individual had a flu vaccination in the past 12 months. Respondents who were smoking at the time of the survey were classified as current smokers. Those who smoked before but were not smoking at the time of the survey were classified as former smokers.

#### Measures of costs

We look at two types of costs associated with limited health literacy:

1. Out-of-pocket health expenditure. A respondent was asked about the out-of-pocket health spending that the respondent incurred within the past 12 months (including the amount paid for medicine but excluding the amount that had been or would be reimbursed by health insurance). The original question asked in the survey is presented in Table A2 in the Supplementary file.
2. Absenteeism due to illness. Respondents were asked whether they had missed work/study or taken a break due to illness in the past 12 months, and the number of days of absenteeism.

### Statistical analyses

Our main outcome variable is out-of-pocket health expenditure. In most populations, medical expense variables at the individual level have a highly skewed distribution: a significant number of respondents do not use medical care and thus have no expenditure, resulting in many “zero” values. Among the nonzero realisations, many use small amounts, and a few individuals incur substantial medical expenses that account for a large percentage of aggregate spending [31]. That is to say, if we use standard statistical methods that assume the dependent variable is normally distributed, we will get inaccurate predictions of costs.

To address the non-normal distribution, we use the two-part model (2 PM) to estimate the out-of-pocket health expenditure associated with limited health literacy. We estimate the first part (whether an individual incurs any medical expenditure) using the logit model and estimate the second part (how much to spend among medical users) using the generalized linear model (GLM) with a gamma distribution and a log link. The model specification is justified by the modified Park test, which shows that the conditional variance function of the medical expenditure distribution is consistent with the gamma class model.^5^ In addition, the result of the Pregibon’s Link Test also confirms that our choice of log link function is consistent with the data generating process.^6^ Following the convention, the same set of control variables are included in two regressions, including rural/urban classification of residence, age, gender, marital status, number of household members, annual household income per capita (log), education level, and job status. We also include self-reported health status variable to net out the effect of health on health literacy following [31].

Our primary interest is not the coefficients but predicting the level of expected out-of-pocket health expenditure that varies with the level of health literacy. It is constructed by computing predicted probabilities from the first stage, multiplying them by the second stage predicted values and averaging the predicted spending levels over the entire sample. Two values are used to summarise the effect of health literacy on spending. The first is the expected level of out-of-pocket health spending with the health literacy variable set equal to zero for every respondent; the second is the expected level of spending with the health literacy variable set equal to one. The two values thus summarise the expected level of out-of-pocket health expenditure conditional on the sample having adequate (or inadequate) health literacy. Computed values in this way net out the impact of observed individual characteristics, such as age, on out-of-pocket spending. All statistical analyses are performed using StataCorp LLC’s Stata Statistics Software version 15.0 [35].

## Results

### Sample characteristics

We examine the characteristics of our respondents by presenting the summary statistics of variables in Table 1. We report mean and standard deviation for continuous variables; percent, and frequency (highlighting the number of the corresponding category) for categorical variables in the first two columns.

**Table 1:** Sample characteristics

Variables	Mean/Percent	S.D./Freq.	Level of HL		
			Adequate	Inadequate	
Observations	*N* = 6316		*N* = 1376	*N* = 4940	
Adequate health literacy (HL) (0/1)	21.8%	*n* = 1376	/	/	/
**Costs**					
Incur OOP health exp. (0/1)	73.6%	*n* = 4647	68.4%	75.0%	***
Nonzero OOP health exp. (1000 CNY)	2.7	9.1	2.1	2.9	***
Absenteeism due to illness (0/1)	7.0%	*n* = 443	8.6%	6.6%	***
Nonzero days of absenteeism last year	30.0	70.2	21.0	33.3	*
**Demographic characteristics**					
Urban (0/1)	62.1%	*n* = 3922	74.3%	58.7%	***
Male (0/1)	48.2%	*n* = 3047	48.0%	48.3%	
Age in years	49.1	13.6	41.9	51.1	***
1:15–44	34.7%	*n* = 2194	60.0%	27.7%	***
2:45–59	37.7%	*n* = 2383	28.6%	40.3%	***
3:60–69	27.5%	*n* = 1739	11.4%	32.0%	***
Married (0/1)	82.0%	*n* = 5180	80.5%	82.4%	
Household size	2.9	1.2	3.1	2.8	***
Household annual inc. pc (1000 CNY)	38.6	46.5	51.6	35.0	***
*Education*					
1:Primary or lower	30.0%	*n* = 1893	9.2%	35.8%	***
2:Middle/High school	47.8%	*n* = 3021	42.7%	0.493	***
3:College or higher	22.2%	*n* = 1402	48.1%	0.150	***
*Job status*					
1:Public sectors	13.1%	*n* = 830	23.3%	10.3%	***
2:Farmers	26.9%	*n* = 1701	9.2%	31.9%	***
3:Manual workers	19.1%	*n* = 1207	14.9%	20.3%	***
4:Private sectors	28.3%	*n* = 1785	43.3%	24.1%	***
5:Other	12.6%	*n* = 793	9.3%	13.5%	***
**Health status**					
Self-reported good health (0/1)	64.4%	*n* = 4068	70.6%	62.7%	***
Any chronic diseases (0/1)	25.0%	*n* = 1579	14.0%	28.1%	***
Cardio-Cerebrovascular diseases (0/1)	19.6%	*n* = 1241	10.1%	22.3%	***
Diabetes (0/1)	5.2%	*n* = 331	2.3%	6.1%	***
*BMI status*					
1:Underweight (< 18.5)	5.7%	*n* = 343	6.7%	5.4%	**
2:Normal (18.5–24)	62.2%	*n* = 3757	66.2%	61.1%	***
3:Overweight (24–28)	27.2%	*n* = 1642	23.9%	28.1%	***
4:Obese (28+)	4.9%	*n* = 298	3.2%	5.4%	***
**Health behaviour**					
*Smoking status*					
1:Never	70.7%	*n* = 4465	76.7%	69.0%	***
2:Quit	8.0%	*n* = 505	5.9%	8.6%	***
3:Smoke	21.3%	*n* = 1346	17.4%	22.4%	***
Flu vaccination (0/1)	1.7%	*n* = 106	1.1%	1.8%	**

Notes: (1) OOP health exp., Out-of-pocket health expenditure; Household annual inc. pc, Household annual income per capita. (2) The sample size for BMI status variable is 6040. (3) ∗ *p* < 0.10, ∗∗ *p* < 0.05, ∗∗∗ *p* < 0.01. The *p*-value is calculated using either the *t*-test (if continuous) or the proportion test (if binary); a pre-test of equality of variance is also conducted. Source: National Health Literacy Surveillance (NHLS) survey in Ningbo, 2019

About 22% of the respondents are identified as having adequate health literacy, above the 2019 national health literacy rate of 19.2% [36]. About 74% had nonzero out-of-pocket health spending, and among those who incurred out-of-pocket expenses, the average amount is 2746 CNY (on 2020-07-10, 1.00 USD = 7.00 CNY, equivalent to 392 USD). These statistics are comparable with what we find in a similar survey China Family Panel Studies (CFPS) that represents adults aged 16 or older. In 2018 CFPS, 81% incurred nonzero out-of-pocket health spending, and the average amount is 2692 CNY (385 USD). Regarding the number of days missed due to illness, about 7% (*N* = 440) had reported absenteeism due to illness. Among those with absenteeism, the average number of days missed is 30 days.

Next, we look at the demographic characteristics. Over 60% of the 6316 respondents are urban residents, in line with the national urbanization rate in 2018 and 2019 [37]. Less than half are men, which by male-to-female sex ratio is 100:108. A typical respondent is 49-years-old, married (82%), and lives with two other members (e.g., spouse and/or children). The average household annual income is 38,600 CNY (5514 USD) per capita after adjusting for household size. This figure is higher than the 2019 national average 30,733 CNY (4390 USD) [38]. In terms of education level, 30% of the respondents have no education or just finished elementary school (low), 48% finished middle or high school (middle), and the rest 22% have a college or higher degree (high). In terms of job status, about 13% are in public sectors, 27% are farmers, 19% are manual labourers, and 28% are working in private sectors.

Next, we move on to examine health outcome measures. Overall, more than half of the respondents reported their health, in general, to be ‘good’ or ‘excellent.’ A quarter of respondents reported having at least one type of chronic disease. The most prevalent type is cardio-cerebrovascular diseases (20%) (including heart problems and cerebrovascular diseases), followed by diabetes (5%).^7^ About 62% of our respondents have normal BMI. The remaining 38% include those being underweight (6%) and overweight (or obese) (32%). The proportion of current smokers is about 21%, and less than 2% of our respondents reported having had a flu vaccination within the past 12 months.

We also split our sample by their level of health literacy and report the respective mean or percentage statistics in the last three columns along with the level of significance associated with the test for the equality of the means (or proportions) between the two samples. These tests help us identify the characteristics associated with limited health literacy. Compared to those with ‘adequate health literacy,’ those with ‘inadequate health literacy’ are on average more likely to incur out-of-pocket health spending, and the average amount is also greater. However, people with ‘inadequate health literacy’ are significantly less likely to report absenteeism due to illness, which we will examine later by looking at its distributions. On average, residents with ‘inadequate health literacy’ are more likely to live in rural areas, older (51 vs. 42), live with fewer household members, and are poorer in annual household income per capita. In addition, they are less educated and more likely to be farmers or manual workers (less likely to work in public sectors or employed in private sectors). Not surprisingly, respondents with ‘inadequate health literacy’ on average have poorer health. Their self-reported health level is lower on average. Both the prevalence rates for chronic diseases and the proportion of underweight and overweight (measured by BMI) are higher among those with ‘inadequate health literacy’, who are also more likely to be current smokers. It appears the rate of flu vaccination is significantly higher among the group with ‘inadequate health literacy’ (1.8% vs. 1.1%). However, the rate of taking flu vaccination is low for the full sample, so it is difficult to judge whether this difference is meaningful.

We also examine the summary statistics of the 138 observations excluded from the analytic sample and find they are not systematically different from those in our analytic sample. These results are reported in Table A3 in the Supplementary file. No significant differences arise in the level of health literacy and the likelihood of incurring any out-of-pocket health expenditure. Compared with our analytic sample, the excluded respondents have similar demographic characteristics regarding rural-urban classification of residence, sex ratio, economic status, and health status. The excluded respondents, however, are on average younger, better educated, more likely to be working in public sectors and less likely to be farmers. Probably due to a small sample size, the excluded observations have a higher proportion of being absent due to illness and having had a flu vaccination in the past 12 months.

### Distribution of nonzero out-of-pocket health spending

**Fig. 1:**
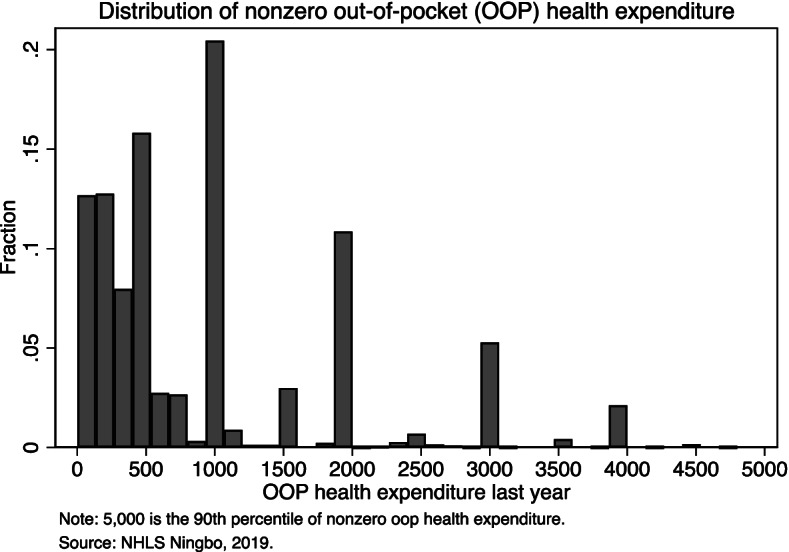
Distribution of nonzero out-of-pocket health expenditure

In Table 1, we find nonzero out-of-pocket medical spending is on average 2746 CNY (392 USD). The median, however, is 1000 CNY (143 USD). Due to its non-normality, this mean statistic does not describe the out-of-pocket health spending of a typical individual. To better understand this expenditure variable, we examine the distribution of out-of-pocket medical spending in our sample by plotting its histogram in Fig. 1. Among the 4647 observations (73% of the full analytic sample) with nonzero out-of-pocket health spending, the 10th, 20th, to 90th percentiles are 100, 200, 500, 500, 1000, 1000, 2000, 2600, 5000 CNY. We plot this graph for only values below the 90th percentile (5000 CNY) to make the histogram more compact. In line with medical spending found in most samples, we have a highly skewed distribution where many individuals use small amounts. There are spikes of values that are multiples of 500, e.g., 500, 1000, and 1500, implying most of these self-reported numbers are not precise numbers.

**Fig. 2:**
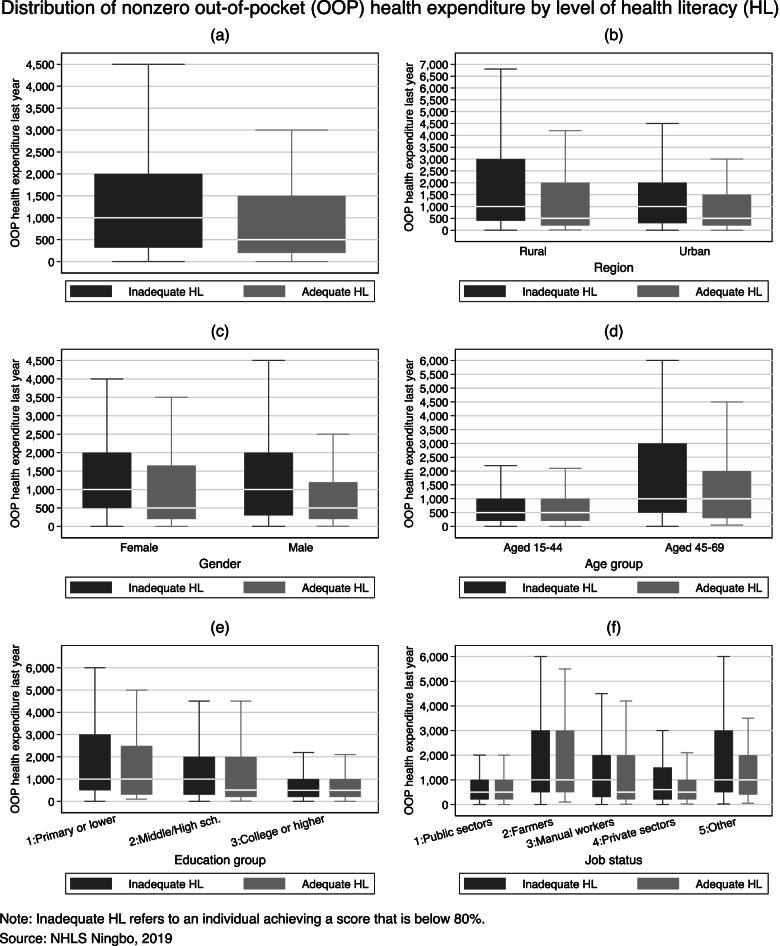
Distribution of nonzero out-of-pocket health expenditure by level of health literacy

Next, we compare nonzero out-of-pocket health expenditure by the level of health literacy using box plots in Fig. 2. The box plot displays box(es) bordered at the 25th and 75th percentiles of nonzero out-of-pocket health spending with a median line at the 50th percentile. Whiskers extend from the box to the upper and lower adjacent values and are capped with an adjacent line. In plot (a) in Fig. 2, we show the median of nonzero out-of-pocket expenses is higher among those with ‘inadequate health literacy’. It suggests that limited health literacy is associated with higher out-of-pocket health spending among medical users. In the next couple of plots in Fig. 2, we break down the distributions by the respondents’ region, gender, age, education, and job status.

In plot (b), we find the same median difference by the level of health literacy in rural and urban samples, respectively. In addition, if we compare the 75th percentile instead of the median, the difference by the level of health literacy is greater in the rural sample than in the urban sample. This disparity might capture the difference in health status between rural and urban residents, leading to differences in the health utilisations and consequent health spending. In plot (c), we find a similar median gap for female and male samples, respectively. In plot (d), we break down the distributions by the age of the respondent, and the gap by the level of health literacy diminishes for each age group. Older people, on average, spend more on health care, but health literacy does not drive the median out-of-pocket health spending differently for the younger or the older group. What we observe in plot (d) suggests that the gap we observed in plot (a) could be driven by the age difference in people with different levels of health literacy. For the older group, however, the bottom and upper quartiles are higher for those with ‘inadequate health literacy’. In plot (e), we break down the distributions by the level of education. Overall, the out-of-pocket health spending is higher for those with a ‘low’ level of education, but there is no median gap by level of health literacy except for the group with a ‘middle’ level of education. In plot (f), we find the gap by the level of health literacy is partly explained by the difference in job status and the gap by the level of health literacy is only observed for the group of manual labourers.

We also use box plots to examine the distribution of the number of days missed due to illness (recall period of the past year) and its relationship with limited health literacy. Among the 443 observations, the reported days of absenteeism has a long tail. Thus, we rescale this variable by taking the logarithm and the results are shown in Fig. A1. The median of days in absenteeism (log) is higher in the group with ‘inadequate health literacy’, as shown in plot (a). When we break down the distribution by rural/urban residence, the median difference due to inadequate health literacy is positive in rural areas but negative in urban areas. We observe the the gap for both genders in plot (c), the older group (aged 45+) in plot (d), the group with a ‘low’ level of education in plot (e), and the group of farmers/manual workers in plot (f).

### Two-part model (2 PM)

Results from the two-part model (2 PM) are presented in Table 2. According to the first part of the 2 PM for out-of-pocket health spending (column 1), adults with ‘adequate health literacy’ are less likely to incur nonzero out-of-pocket medical expenditure by two percentage points (*p <* 0.10). Among those who incurred nonzero out-of-pocket medical expenses, however, the amount does not differ by health literacy level (column 2). In other words, the role of health literacy is more relevant in the first stage when an individual considers whether or not to seek health care. Similar results are found in [2], where persons with inadequate health literacy are more likely to use inpatient services, but among those who used inpatient care, spending does not differ by health literacy status.

**Table 2:** Two-parts model of out-of-pocket health spending

	(1)		(2)	
	Participation (mar. eff.)		Intensity (mar. eff.)	
Adequate health literacy (0/1)	−0.023*	(0.013)	−180.602	(302.692)
Urban (0/1)	−0.030***	(0.012)	113.433	(257.977)
Male (0/1)	−0.010	(0.011)	54.884	(251.383)
*Aged 15–44*	ref.		ref.	
45*–*59	−0.022	(0.015)	646.878**	(297.995)
60*–*69	0.004	(0.019)	1092.308***	(388.938)
Married (0/1)	0.016	(0.015)	419.981	(311.173)
*Education (1:Low)*	ref.		ref.	
2:Middle/High sch.	−0.036**	(0.015)	− 713.841**	(344.405)
3:College or higher	−0.081***	(0.022)	− 766.065	(481.152)
Public/Private sectors (0/1)	−0.024*	(0.014)	− 391.532	(297.586)
Household size	0.009*	(0.005)	162.790	(111.224)
Annual hh inc. pc (log)	−0.011***	(0.004)	−92.940	(88.939)
*Self-report health (1:very good)*	ref.		ref.	
2:good	0.129***	(0.015)	460.662**	(189.925)
3:average	0.210***	(0.015)	1889.934***	(276.224)
4:poor	0.325***	(0.021)	7145.947***	(1745.329)
5:very poor	0.324***	(0.037)	10,972.468**	(5037.355)
Dep Mean	0.736		2745.921	
Pseudo R^2^	0.060		/	
AIC	6885		80,875	
Obs	6316		4647	

Notes: (1) The reported statistics are the average marginal effects. (2) The dependent variable in column 1 is binary, indicating whether a respondent incurred any out-of-pocket health spending in the last 12 months. The dependent variable in column 2 is the level of out-of-pocket health spending for the sample with nonzero expenses. (3) Adequate health literacy is measured by having 80% score to the health-related skills questions. (4) Standard errors in parentheses

^∗^*p <* 0.10

^∗∗^*p <* 0.05

^∗∗∗^*p <* 0.01

The coefficients of other control variables are generally consistent with the current literature on the demand for health care and align with our descriptive analyses in Table 1. Specifically, we find urban residents are less likely to incur nonzero out-of-pocket health spending. The likelihood and the level of out-of-pocket health spending decrease with education. There is a large gap in out-of-pocket spending between different age groups. For example, the group aged 60–69 on average has a higher expenditure by 1092 CNY (156 USD) relative to those aged 15–44.^8^ It seems that respondents from larger households are more likely to incur out-of-pocket health spending, and the level is also higher. Annual household income per capita turns out to be negatively associated with the likelihood of incurring out-of-pocket health spending, suggesting that those with a better financial capacity to pay are less likely to use any medical services. Lastly, health status matters and is a key driver. Those who self-reported poorer health are more likely to incur out-of-pocket health spending. For example, compared to those who self-reported a level of health to be ‘very good’, those who reported ‘very poor’ on average incur 10,972 CNY (1567 USD) more on medical expenses.

Based on the regression results of the 2 PM, we present the predicted expected medical cost attributed to limited health literacy for the full sample and the subsamples by rural/urban area, gender, and age in Table 3. We report for each subsample: (a) the predicted expected individual out-of-pocket medical spending; (b) the counterfactual expected out-of-pocket medical expenditure when the level of health literacy is set to ‘inadequate’; (c) the counterfactual expected out-of-pocket medical expenditure when the level of health literacy is set to ‘adequate’; (d) the expected medical costs of limited health literacy, i.e., the difference in (b) and (c); (e) in percentage terms the share of this difference in the expected medical spending; and (f) the *t* statistics and *p* values associated with the *t-*tests on the difference of cost estimates in (b) and (c).

**Table 3:** In-sample predicted expected out-of-pocket health expenditure for adults with ‘adequate’ and ‘inadequate’ health literacy based on the 2 PM, NHLS Ningbo, 2019

Sample	Obs	(1)	(2)	(3)	(4)	(5)	(6)	(7)
		Exp. exp.	Inadequate HL	Adequate HL	Diff	Diff (%)	*t*-stat	*p*-value
Baseline	6316	2053.44	2085.25	1908.67	176.58	9.68	−91.67	0.000
Rural	2394	2383.61	2426.08	2077.21	348.86	15.66	−52.33	0.000
Urban	3897	1784.77	1810.01	1699.33	110.67	7.19	−76.62	0.000
Female	3269	2060.59	2101.96	1866.53	235.43	12.37	−66.61	0.000
Male	3047	2038.98	2070.26	1899.23	171.03	9.64	−56.77	0.000
Young	1675	1017.43	1061.95	940.77	121.18	12.15	−48.11	0.000
Old	4640	2414.93	2447.91	2228.88	219.04	10.54	−93.34	0.000

Notes: (1) All subsample results are based on the same two-part model (2 PM). (2) The counterfactual out-of-pocket medical expenditure in columns 2–3 are computed by setting the level of health literacy to ‘inadequate’ (or ‘adequate’) for the corresponding sample, holding other individual characteristics at the actual levels. (3) The *t* statistics and *p* values are associated with the *t-*test on the significance of the cost estimates (the difference between the two counterfactual expenditures in columns 2–3). (4) Age groups are based on the following classification: ‘young’ = aged 15–39 and ‘old’ = aged 40–69

The annual expected out-of-pocket health expenditure due to limited health literacy is predicted to be 177 CNY (26 USD), or around 10% of the total expected out-of-pocket health spending of 2053 CNY (293 USD) in a year. A comparison among subsamples suggests that the individual medical costs of limited health literacy are not evenly distributed across regions and subpopulations, with the rural residents, females, older people bearing greater medical costs due to limited health literacy. For example, the medical cost of limited health literacy is larger in the rural areas (349 CNY or 50 USD) than that in the urban areas (111 CNY or 16 USD). Not only in amount, but rural residents also bear a greater burden because they pay a greater share for limited health literacy (16% vs. 7%). Similarly, we find the cost of limited health literacy in out-of-pocket medical expenditure is higher for females and for the older group.

### Estimates of aggregate medical costs of limited health literacy

The 2 PM estimates allow us to provide estimates of the aggregate medical costs for different populations with different levels of health literacy. These estimates will help us to justify the cost of relevant public policies that aim to improve health literacy. A 2013 WHO report shows “limited health literacy cost more than US $8 billion, an estimated 3–5% of the total health care budget in Canada in 2009. In 1998, the United States National Academy on an Aging Society estimated that the additional health care costs caused by limited health literacy were about US $73 billion.” [40]. There are no comparative figures in China, so we aim to provide a preliminary estimate of this cost.

First of all, it is helpful to have some ideas about the comparability of our medical costs with actual numbers from other sources. According to our 2019 NHLS survey in Ningbo, the self-reported out-of-pocket health spending per capita is 2746 yuan (392 USD), and the adjusted estimate from our 2 PM estimation is slightly lower, at 2053 yuan (293 USD). From the most recent China Health Statistics Yearbook, the 2017 actual health expenditure per capita in Zhejiang and in China is 4996 CNY (714 USD) and 3784 CNY (541 USD), respectively [41]. The proportion of out-of-pocket health expenditure in Zhejiang province is 26.99%, thus the actual amount of out-of-pocket health expenditure per capita in Zhejiang province is 1348 CNY (193 USD) [41]. Similarly, the proportion of out-of-pocket health expenditure in China is 28.91%, so the actual amount of out-of-pocket health expenditure per capita for the country is 1089 CNY (156 USD) [41, 42]. Although these figures are aggregated across the population covering all age groups, whereas our data is representative of residents aged 15–69, our per capita estimates are largely comparable with the actual per capita figures (it is robust after converting our figures to ones in 2017 price).

We aimed to compute the aggregate health expenditure for the adult population aged 15–69 in Ningbo with different levels of health literacy. We used the adult population aged 16+ instead on the grounds that we can only obtain the data for the population aged 16+, and this population is close to what our sample (aged 15–69) represents. Two estimates from the 2 PM are used: the expected out-of-pocket health expenditure per capita is 2085.25 CNY (298 USD) for an individual aged 15–69 with ‘inadequate health literacy’ and 1908.67 CNY (273 USD) for an individual aged 15–69 with ‘adequate health literacy’. We assume the expected expenditure does not change with other characteristics of an individual except for the level of health literacy. Thus, we could compute the total out-of-pocket health expenditure in a population with a specific level of health literacy as follows:

1 $$(2085.25\times {POP}_0)+\left(1908.67\times {POP}_1\right)$$

where *POP*_0_ refers to the number of people with ‘inadequate health literacy’, *POP*_1_ refers to the number of people with ‘adequate health literacy’. For example, for Ningbo, given the adult population (aged 16+) is around 7.47 million in 2019 [19] and the proportion of residents with ‘adequate health literacy’ is 22% in 2019, we can compute the number of people with and without adequate health literacy (i.e., *POP*_0_ and *POP*_1_). The number of people with adequate health literacy would be 1.64 million (i.e., 7.47 million × 22%), and the number of people with inadequate health literacy would be 5.83 million (i.e., 7.47 million × 78%). The total out-of-pocket health expenditure for Ningbo would be 15.28 billion CNY (2.18 billion USD) using formulae (1). We further estimate aggregate health expenditure at other levels of health literacy, and the results are reported in Table 4.

**Table 4:** Estimates of the aggregate out-of-pocket (OOP) health expenditure that changes with the level of health literacy

	Adult Population (million)	Proportion of people with adequate health literacy					
		22%	30%	40%	50%	60%	70%
		Total OOP Health Expenditure (billion CNY)					
Ningbo	7.47	15.28	15.18	15.05	14.92	14.78	14.65
Zhejiang	38.20	78.17	77.63	76.96	76.28	75.61	74.93
China	896.40	1834.40	1821.73	1805.90	1790.07	1774.25	1758.42

Notes: (1) The adult population (aged 16+) for Ningbo, Zhejiang, and China is 7.47 million, 38.20 million, and 896.40 million, respectively, end of the 2019 year, which we use to construct the aggregate OOP health expenditure. (2) The total OOP health expenditure for a population is computed by using 2085.25 × *POP*_0_ + 1908.67 × *POP*_1_, where *POP*_0_ refers to the number of people with inadequate health literacy, *POP*_1_ refers to the number of people with adequate health literacy. For example, if the level of health literacy is 30% among the adult population in Ningbo, other things being equal, the number of people with adequate health literacy would be 2.24 million (7.47 million × 30%) and the number of people with inadequate health literacy would be 5.23 million (7.47 million × 70%), which we use for the values of *POP*_0_ and *POP*_1_. The total OOP health spending for the adult population at a level of health literacy of 30%, thus, would be 15.18 billion CNY in Ningbo

If the proportion of people with ‘adequate health literacy’ increases to 30%, other things being equal, the total out-of-pocket health expenditure would decrease from 15.28 billion CNY to 15.18 billion CNY. In other words, 100 million CNY (14 million USD) can be saved. This aggregate expenditure can be further reduced to 14.65 billion CNY (2.09 billion USD) if the proportion of people with ‘adequate health literacy’ reaches 70%. Similarly, we use the adult population (aged 16+) for Zhejiang (38.20 million in 2019) and for China (896.40 million in 2019) to estimate the corresponding aggregate out-of-pocket health expenditures. These results are presented in the next two rows in Table 4. These numbers are for illustration and we discuss their implications later.

## Discussion

With a selected sample from the NHLS survey conducted in Ningbo, a coastal city in Eastern China, we study the medical costs associated with limited health literacy using the 2 PM model. Our estimation using the 2 PM model is built on examining the summary statistics of our sample and the distribution of out-of-pocket health expenditure reported by our respondents. We then used the estimates from the 2 PM model to compute the medical cost of limited health literacy at the individual level and for the relevant populations.

We define a respondent as having ‘inadequate health literacy’ (thus limited health literacy) if the respondent obtained a score below 80% to the NHLS questions following the same threshold used by the government. We show among adults aged 15–69 living in Ningbo, about 78% of residents are classified as having ‘inadequate health literacy’ in 2019, which is below the national rate of 81% in the same period [43]. Although the level is lower in Ningbo, inadequate health literacy is quite common.

The descriptive analyses in Table 1 show limited health literacy is associated with poorer health outcomes in terms of self-reported health status and chronic diseases. We use box plots in Fig. 2 to examine the distributions of nonzero out-of-pocket health expenditure by the level of health literacy and find a gap in median out-of-pocket health expenditure. This gap persists when we examine the distribution of out-of-pocket health expenditure for subsamples by region or gender and is found among adults with a middle-level education (i.e., completed middle or high school) and manual workers. This health literacy-induced gap, however, diminishes in the subsamples by age and job status, two characteristics that are strongly correlated with the level of health literacy. Despite this, if we compare the upper quartile (75th percentile) instead of the median, the health literacy-induced gap is more likely to be observed, implying that better health literacy might protect individuals from large health expenditure, preventing them from experiencing poverty due to large health expenditure.

We then use the 2 PM to examine the out-of-pocket health expenditure associated with limited health literacy to net out the effect from demographic and socioeconomic factors. We find limited health literacy is associated with an increase in expected out-of-pocket health expenditure by 177 CNY (25 USD), or 10% of an individual’s expected out-of-pocket health expenditure. This 177 CNY (25 USD) may appear to be a small number in absolute terms, given that an average resident living in Ningbo spent 2223 CNY (318 USD) on medicine and medical services in 2019 [19].^9^ In addition, this number cannot be compared with other studies on medical costs of limited health literacy because the sample and the measurement of health literacy are often different.^10^ In comparison to studies on the medical costs of other adverse health conditions, however, this 10% indicates that ‘inadequate health literacy’ is very costly to individuals and society as a whole. For example, a study on the medical costs of overweight and obesity in China found that the per capita medical cost attributable to obesity and overweight was estimated to take 5% of the total personal medical expenditure from 2000 to 2009 [44]. Another study examining the medical costs of depression in China find about 7% of the personal medical expenditure among Chinese adults was attributed to depression [45]. We also show the cost of limited health literacy is greater for rural residents than for the urban residents expressed in level and percentage. The corresponding figures are 348 CNY (16%) and 110 CNY (7%), for rural and urban subsamples, respectively. Disparities also arise across genders, and the medical cost of limited health literacy is greater for females than for males.

With the 2 PM estimates, we further assess the aggregate out-of-pocket health expenditure that varies with the level of health literacy for the adult population (aged 16+) in Ningbo, which to our knowledge, is the first study of its kind in China. We show if the proportion of people with ‘adequate health literacy’ increases from 22% (the actual level in 2019) to 30% (the level to be achieved by 2030 according to the 2030 Healthy China Framework), 100 million CNY (14 million USD) would be saved. This number also helps us justify the cost of a health literacy intervention program. If the program can improve the rate of health literacy to 30% in the population and the monetary cost is lower than 100 million CNY (14 million USD), then the cost of the program is justified. We also look at this number in the context of the government fiscal expenditure. In 2019, the fiscal expenditure in Ningbo was 176,790 million CNY (25,256 million USD), among which the expenditure on health care was 11,400 million CNY (1629 million USD) [19]. A 100 million CNY (14 million USD) amounts to 0.88% of Ningbo’s government health care expenditure in 2019.

We acknowledge that the effect of limited health literacy in our subpopulation (residents aged 15–69 living in Ningbo) may not generalize to the adult population (aged 16+) in Ningbo. Because the sample we used excludes those aged 70+, our aggregate estimates are likely to be underestimated as the population aged 70+ are often most at risk of low health literacy and increasing health care costs [46, 47]. Similarly, we could not assume our estimates can be generalized to the adult population (aged 16+) in Zhejiang or China, so the primary focus of this paper is the marginal effect of limited health literacy for our subpopulation rather than the national estimates. The national estimates, however, help us gauge the reliability of our estimates using data from Ningbo. Our estimate for the national total out-of-pocket health expenditure (assuming the level of health literacy is 22%) is around 1834 billion CNY (or 262 billion USD), which is comparable with the actual official figures (1513 billion CNY or 216 billion USD) [41]. In addition to the strong assumptions we made, we also need to partial out the fact that the national level of health literacy (at 19%) is lower than that in Ningbo (at 22%).

Similarly, we use box plots to examine the cost of limited health literacy measured by the number of days of absenteeism due to illness (see Fig. A1). Adults with limited health literacy have more days of absenteeism in a year, and the pattern is strong in the rural subsample, the older subsample, the subsample with a ‘low’ level of education, and the subsample of farmers/manual workers. In contrast, the opposite is true in the urban subsample, where we observe a higher median among those with ‘adequate health literacy’ than those with ‘inadequate health literacy’. Notice we are comparing the number of days of absenteeism among adults who had absenteeism and excluded those who had an illness but did not have any absenteeism. That left us with a relatively small sample, and the sample statistics are less accurate and come with large standard errors.

It is worth noting that we measure the costs using out-of-pocket health expenditure, not total health expenditure (including the amount paid by the health insurance), as did other studies that used patient-level data. It is possible those with low out-of-pocket health spending are those with better health insurance instead of better health literacy. This is not a big problem because the private health insurance in China is relatively low in coverage, and the government-sponsored health insurance programs have near-universal coverage. In China, the social health insurance schemes reached 95% of the national population in 2018 [48]. Nonetheless, it remains the direction for future studies to account for the difference in health insurance.

Another concern is we used the self-reported out-of-pocket health expenditure, which is likely to be subject to recall bias. As it is shown in Fig. 1, there is a high frequency of values of out-of-pocket health spending that peaks around numbers that are multiples of 500. Despite this, this measurement error is unlikely to be systematically correlated with health literacy level for conceivable reasons. Thus, we do not think we should be concerned.

There are several other limitations in our study, and the results should be interpreted with caution. Firstly, our study is built on cross-sectional data. There may be unobserved individual characteristics that are correlated with both health literacy and out-of-pocket medical spending, confounding our estimates. Second, we acknowledge that the effect of limited health literacy in our subpopulation (residents aged 15–69 living in Ningbo) may not generalize to the adult population (aged 16+) living in Ningbo, nor could we assume our estimates can be generalized to the adult population (aged 16+) in Zhejiang or China. Third, our health literacy measurement can be limited in measuring health literacy and is not comparable with measures used in other countries. Some might argue a Cronbach’s alpha of 0.95 is too high, indicating redundancy in the questions being asked. Despite their reliability and validity, we mentioned earlier, the construct instruments included in the questionnaire have been unchanged in the past few years. It implies respondents with a higher health literacy score may not be more knowledgeable but simply better at taking tests than those who achieved lower scores. The choice of the threshold in classifying different levels of health literacy can also be a question for future research. Lastly, we control for health status in our 2 PM model by assuming changes in health affect health literacy. For example, health literacy might be improved in the wake of chronic disease diagnosis [49]. Our estimates remove the effect of health on health literacy and health (e.g., chronic disease prevention), which is likely to underestimate the actual effect systematically.

## Conclusions

To conclude, our results suggest that limited health literacy is costly to individuals and society as a whole. Medical costs of limited health literacy are about 10% (177 CNY or about 25 USD) of the annual medical expenditure of a resident aged 15–69 living in Ningbo. We also find some evidence that having limited health literacy may be associated with more days of absenteeism due to illness in a year. Thus, limited health literacy is associated with a disease burden on individuals, as evidenced by previous studies and high costs in the health system that hurt individuals and society. We conclude limited health literacy is expected to drain China’s health system resources. We estimate the aggregate out-of-pocket health spending that varies with the proportion of people with ‘adequate health literacy’ for the adult population in Ningbo. If the proportion of people with ‘adequate health literacy’ increases from 22 to 30% (the target level by 2030), the aggregate out-of-pocket health expenditure for the adult population (aged 16+) living in Ningbo would decrease by 100 million CNY (14 million USD), or 0.88% of the Ningbo government expenditure on health care in 2019. Our study should help policymakers justify the potential benefit of health literacy intervention programs that aim to improve residents’ health literacy. In addition, considering the uneven distribution of medical costs associated with limited health literacy, our results suggest that policymakers should devote more attention to the disadvantaged groups such as rural residents, which in turn may be an effective way to improve the overall health literacy level and reduce the medical expenditure for the country.

## Supplementary Information

**Additional file 1: Appendix A. Table A1. ** Examples of items in 2019 NHLS survey. **Table A2.** Questions on chronic diseases in 2019 NHLS survey. **Table A3.** Sample characteristics of respondents excluded from analytic sample. **Figure A1.** Distribution of days of absenteeism due to illness by level of HL. **Appendix B.** Questionnaire.

## Data Availability

The data used and/or analysed in the current study are not publicly available because restrictions apply to the availability of the data. Data is, however, available from the corresponding author on reasonable requests and with permission of Ningbo Municipal CDC.

## Abbreviations

HL: Health literacy
OOP: Out-of-pocket
NHLS: National Health Literacy Surveillance
PSU: Primary Sampling Unit
PPS: Probabilities Proportional to Size
WHO/OMS: World Health Organization (Organisation Mondiale de la Santé)

## Footnotes

1 For example, in 2003 National Assessment of Adult Literacy data (the first national representative assessment of adult health literacy in US), it is reported that over one third of adults in US had limited health literacy [9]. In the Program for the International Assessment of Adult Competencies (PIAAC) conducted in 2011/14, it is reported on average US adults aged 16–65 have a level of literacy that is “below basic”, which does not change in the PIAAC conducted in 2017 [10].

2 The share of out-of-pocket helath spending out of annual income is also higher in LMICs than in high-income countries. Using 2018 GDP per capita data from World Bank, we calculated out-of-pocket spending as a share of annual income (GDP per capita) for different income groups [13]. In 2018, the average out-of-pocket spending per capita as a share of GDP per capita was highest for low income countries at 2.44%, followed by 2.13% for lower-middle income countries, 1.72% for upper-middle income countries and 1.54% for upper-middle income countries.

3 To have a clear understanding of where Ningbo is located in China, interested readers can refer to Ningbo China Map at https://www.chinadiscovery.com/zhejiang/ningbo/maps/ningbo-china-map.html.

4 In a later study with a different sample, the validity of the national health literacy scale was validated again. The overall Cronbach’s alpha of the (original) 80 items in the 2012 questionnaire was 0.95, and the Spearman-Brown split-half coefficient is 0.94 [24].

5 Modified Park test is used to determine the family of distribution in GLM model [32]. See [33] (pages 61–65) for the Stata code to conduct the test. The coefficient estimate is 2.039. We can reject gaussian (coefficient of 0), poisson (coefficient of 1), and inverse gaussian distribution (coefficient of 3) at 10% level, but we cannot reject gamma family (coefficient of 2, with a *p*-value of 0.938), suggesting that the expenditure variable can be fitted with a gamma distribution.

6 The Pregibon’s Link test intends to check the goodness of fit of the link function in GLM [34]. See [33] (pages 61–65) for the Stata code to conduct the test. After estimating the model with gamma link function, we calculate the predicted value of the expenditure variable. We then estimate a secondary regression with the same expenditure variable and link function but with the predicted value of expenditure variable and its square terms as the model predictors. If the link function is correctly specified, then the square term should not be significant, suggesting the model is fit. In our model, the quadratic term is insignificant with a *p*-value of 0.168.

7 We group hypertension, heart problems and cerebrovascular diseases into one category called ‘cardio-cerebrovascular diseases’ because hypertension is associated with the strongest evidence for causation among the risk factors for cardiovascular diseases and cerebrovascular diseases [39]. In addition, the prevalence of hypertension is high in the adult population.

8 The age effect is likely to capture the health difference between different age groups or the fact that younger people can navigate the health care system better, thus having less out-of-pocket medical cost. Given that we have both health literacy (a partial proxy for the ability to navigate the health care system) and self-reported health status (a proxy to accocunt for the difference in health) in the regression, we think both explanations can play a role in explaining the age effect on out-of-pocket health spending.

9 According to [19], the average per capita consumption is 38,274 CNY in Ningbo, in 2019. In relative terms, the consumption on medicine and medical services takes about 5.8% of the total consumption. The consumption on food, alcohol and tobacco takes about 27.3% and the consumption on recreation, education and culture takes around 12.3%.

10 In a 2004 study, for example, a substantial gap in medical charges by literacy skills was identified by a factor of 3.7 [15]. The sample used by the study, however, is small and represents relatively a disadvantaged group (a sample of 402 randomly selected Medicaid enrollees in the US).
