# Supplementary material for: The economic costs of limited health literacy in China: evidence from China’s National Health Literacy Surveillance data
Source: BMC Health Serv Res. 2022 Apr 20;22:521. doi: 10.1186/s12913-022-07795-9 (PMC9020016; doi:10.1186/s12913-022-07795-9)
Supplement: Supplementary file 1 — Additional file 1: Appendix A. Table A1. Examples of items in 2019 NHLS survey. Table A2. Questions on chronic diseases in 2019 NHLS survey. Table A3. Sample characteristics of respondents excluded from analytic sample. Figure A1. Distribution of days of absenteeism due to illness by level of HL. Appendix B. Questionnaire. [file 12913_2022_7795_MOESM1_ESM.docx]

## Appendix A

**Table A1** Examples of items in 2019 NHLS survey

| **Type of items** | **Examples** | **Dimension** | **Scope** |
| --- | --- | --- | --- |
| True/false | **A01.** The most effective way to prevent influenza is to take antibiotics (anti-inflammatory drugs). | Knowledge and attitude | Infectious disease prevention |
|  | **A04.** Vegetables and fruits provide nutrients of similar kind, so vegetables can be replaced by fruits in your diet. | Behavior and lifestyle | Chronic disease prevention |
| Single-answer | **B01.** The integrated conception of health is:  (1) Good physical well-being without any illness. (2) Good physical and mental well-being.  (3) Good state of complete physical, mental, and social well-being and not merely the absence of disease or infirmity. (4) I do not know. | Knowledge and attitude | Scientific views of health |
|  | **B23.** In the event of a fire, which one of the following responses is correct:  (1) Encase your head with your arms or clothes and rush out of the fire.  (2) You should not escape by using the elevator.  (3) Evacuate while fighting the flames with clothes.  (4) I do not know. | Health-related skills | Safety and first aid |
| Multiple-answer | **C08.** The benefits of eating soybean products such as tofu and soymilk are:  (1) It is good for your health.  (2) It is good for people with cardiovascular diseases.  (3) It is source of good proteins. (4) It can be used as treatment for certain diseases. (5) I do not know. | Behavior and lifestyle | Chronic disease prevention |
|  | **C11.** When coughing or sneezing, the correct way to do this are:  (1) Cover your mouth and nose directly with your hands. (2) Cover your mouth and nose with a handkerchief or a paper towel. (3) Cover your mouth and nose with your elbows. (4) Do not cover your mouth and nose. (5) I do not know. | Behavior and lifestyle | Infectious disease prevention |
| Vignette question | **D01.** (A paragraph of introduction to body mass index and its calculation is given before the question) Mr. Li is 45 years old with a height of 170 cm and weighs 80 kg. How to calculate his BMI? (single-answer)  (1) (80)^2^ / 170 = 37.6. (2) 80 / (1.7)^2^ = 27.2. (3) 160 / (170)^2^ = 55.4. (4) I do not know. | Health-related skills | Health information |
|  | **D03.** (Same as above) To help Mr. Li control weight, which of the following methods can be used? (multiple-answer)  (1) Do not eat rice. (2) Daily exercise for at least 30 min. (3) Reduce fat intake. (4) Eat only vegetables and fruits. (5) I do not know. | Knowledge and attitude | Chronic disease prevention |

Source: 2019 National Health Literacy Surveillance (NHLS) survey questionnaire.

**Table A2** Questions on chronic diseases in 2019 NHLS survey

|  | **Question** |
| --- | --- |
| Chronic diseases | **F09.** Do you have any of the following chronic diseases? (choose all options that apply)  (1) I have no chronic disease 🡪 Skip to **F11**  (2) Hypertension (3) Heart diseases  (4) Cerebrovascular diseases (e.g., stroke, cerebral ischemic stroke and cerebral embolism)  (5) Diabetes (6) Malignant tumour (7) Other |
| Out-of-pocket health spending | **F17.** In the past 1 year, the out-of-pocket health spending is about ______ yuan (including the amount paid for medicine but excluding the amount that had been or would be reimbursed by health insurance). |

Source: 2019 National Health Literacy Surveillance (NHLS) survey questionnaire in Ningbo.

**Table A3** Sample characteristics of respondents excluded from analytic sample

| Sample | Excluded from analytic sample due to non-response  (N=138) | | | Analytic sample (N=6,316) | *p*-value |
| --- | --- | --- | --- | --- | --- |
| Variables | count | Mean/  Percent | S.D./  Freq. | Mean/  Percent |  |
| Adequate health literacy (HL) (0/1) | 138 | 24.6% | n=34 | 21.8% | 0.423 |
| **Costs** |  |  |  |  |  |
| Incur OOP health exp. (0/1) | 88 | 69.3% | n=61 | 73.6% | 0.369 |
| Nonzero OOP health exp. (1,000 CNY) | 61 | 2.205 | 4.896 | 2.746 | 0.402 |
| Absenteeism due to illness (0/1) | 138 | 21.0% | n=29 | 7.0% | 0.000 |
| Nonzero days of absenteeism last year | 7 | 30.000 | 74.978 | 29.991 | 1.000 |
| **Demographic characteristics** |  |  |  |  |  |
| Urban (0/1) | 138 | 61.6% | n=85 | 62.1% | 0.904 |
| Male (0/1) | 135 | 53.3% | n=72 | 48.2% | 0.242 |
| Age in years | 133 | 46.466 | 14.949 | 49.077 | 0.048 |
| 1:15–44 | 133 | 48.1% | n=64 | 34.7% | 0.001 |
| 2:45–59 | 133 | 25.6% | n=34 | 37.7% | 0.004 |
| 3:60–69 | 133 | 26.3% | n=35 | 27.5% | 0.756 |
| Married (0/1) | 135 | 84.4% | n=114 | 82.0% | 0.466 |
| Household size | 133 | 2.895 | 1.110 | 2.887 | 0.940 |
| Household annual inc. pc (1,000 CNY) | 107 | 35.798 | 33.701 | 38.597 | 0.400 |
| *Education* |  |  |  |  |  |
| 1:Primary or lower | 133 | 20.3% | n=27 | 30.0% | 0.016 |
| 2:Middle/High school | 133 | 46.6% | n=62 | 47.8% | 0.781 |
| 3:College or higher | 133 | 33.1% | n=44 | 22.2% | 0.003 |
| *Job status* |  |  |  |  |  |
| 1:Public sectors | 131 | 25.2% | n=33 | 13.1% | 0.000 |
| 2:Farmers | 131 | 19.1% | n=25 | 26.9% | 0.045 |
| 3:Manual workers | 131 | 14.5% | n=19 | 19.1% | 0.184 |
| 4:Private sectors | 131 | 27.5% | n=36 | 28.3% | 0.844 |
| 5:Other | 131 | 13.7% | n=18 | 12.6% | 0.686 |
| **Health status** |  |  |  |  |  |
| Self-reported good health (0/1) | 132 | 66.7% | n=88 | 64.4% | 0.592 |
| Any chronic diseases (0/1) | 135 | 26.7% | n=36 | 25.0% | 0.658 |
| Cardio-Cerebrovascular diseases (0/1) | 135 | 18.5% | n=25 | 19.6% | 0.744 |
| Diabetes (0/1) | 135 | 5.2% | n=7 | 5.2% | 0.977 |
| *BMI status* |  |  |  |  |  |
| 1:Underweight (<18.5) | 117 | 6.8% | n=8 | 5.7% | 0.592 |
| 2:Normal (18.5-24) | 117 | 70.9% | n=83 | 62.2% | 0.053 |
| 3:Overweight (24–28) | 117 | 19.7% | n=23 | 27.2% | 0.069 |
| 4:Obese (28+) | 117 | 2.6% | n=3 | 4.9% | 0.239 |
| **Health behaviour** |  |  |  |  |  |
| *Smoking status* |  |  |  |  |  |
| 1:Never | 133 | 72.2% | n=96 | 70.7% | 0.709 |
| 2:Quit | 133 | 5.3% | n=7 | 8.0% | 0.249 |
| 3:Smoke | 133 | 22.6% | n=30 | 21.3% | 0.729 |
| Flu vaccination (0/1) | 113 | 4.4% | n=5 | 1.7% | 0.026 |

Notes: (1) OOP health exp., Out-of-pocket health expenditure; Household annual inc. pc, Household annual income per capita. (2) The sample size for BMI status variable is 6,040. (3) The *p*-value is calculated using either the *t*-test (if continuous) or the proportion test (if binary); a pre-test of equality of variance is also conducted. Source: National Health Literacy Surveillance (NHLS) survey in Ningbo, 2019.

**Figure A1 Distribution of days of absenteeism due to illness by level of HL**

## Appendix B

Questionnaire code:

National Health Literacy Surveillance survey in Zhejiang Province

Hello! The National Health Commission is conducting the national health literacy surveillance survey. The purpose of the survey is to understand the health knowledge and skill level of Chinese residents, and you have been selected to participate in this survey. Your participation is very important to us. Your information and your answers will be kept strictly confidential and will have no adverse effect on any individuals. The survey results are an important basis for evaluating the health literacy level of Chinese residents and for formulating health-related policies.

If you would like to participate in this survey, please sign ________, date ________.

Thank you for your support and cooperation!

Investigator: _______ Date of Investigation: ________

Quality Control: ________ Verification Date: ________

To be filled by the investigator:

Questionnaire completion status: ① Completed by oneself

② Face-to-face interview because the respondent has a low educational level

③ Face-to-face interview for other reasons (please specify: ________)

________ province ________ city _______ county (district)

________ community (township) ________ subdivision (village)

**1. True or False. (please tick “√” in parentheses if you think the statement is true, and “×” if you think it is false)**

**A01.** The most effective way to prevent influenza is to take antibiotics (anti-inflammatory drugs). ( )

**A02.** Healthcare food is not medicine, nor can it replace medicine to treat diseases. ( )

**A03.** Infusion (intravenous drip) has good and quick effect, so when you are sick, you should choose infusion as your first choice. ( )

**A04.** Vegetables and fruits provide nutrients of similar kind, so vegetables can be replaced by fruits in your diet. ( )

**A05.** The normal body temperature can fluctuate within a day, but the range generally does not go beyond 1℃. ( )

**A06.** Children and adolescents can experience depression. ( )

**A07.** As the saying goes “prolonged illness makes the patient a good doctor”, patients with chronic diseases can adjust their treatment plan according to their own feelings. ( )

**A08.** For problems and diseases detected in the health check-up, before symptoms arise, one may not take any serious action to the problems. ( )

**2. Multiple-choice questions with a single answer. (only 1 of the 4 options is correct for each question, tick “√” on the corresponding option number you think is correct. If you do not know, please select 4)**

**B01.** The integrated conception of health is:

① Good physical well-being without any illness. ② Good physical and mental well-being.

③ Good state of complete physical, mental, and social well-being and not merely the absence of disease or infirmity. ④ I do not know.

**B02.** Hepatitis B can be transmitted to others in one of the following ways:

① Contact with a patient or an infected person via working, eating, or swimming together.

② Can be transmitted via sex, blood transfusion, mother-to-child transmission.

③ When talking, shaking hands, and hugging with a patient or an infected person.

④ I do not know.

**B03.** Regarding the self-monitored blood pressure, which of the following statements is incorrect?

① Self-monitored blood pressure can help doctors’ diagnosis.

② Patients with high blood pressure need to self-monitor their blood pressure regularly, and the records can be used as reference by doctors to design treatment plan and to assess the outcomes of the treatment.

③ Patients with high blood pressure can be exempted from outpatient visits for regular follow-up check if their self-monitored blood pressure reads stable.

④ I do not know.

**B04.** Regarding the danger of smoking, which of the following statements is incorrect:

① Tobacco dependence is a chronic addictive disease.

② Smoking can cause several chronic diseases.

③ Low-tar cigarettes are less harmful than regular cigarettes.

④ I do not know.

**B05.** Which one is not an early warning sign of cancer:

① Abnormal lumps in your body. ② Blood in your stool.

③ Weight increases for no known reasons. ④ I do not know.

**B06.** In case of gas poisoning, how should the rescuer deal with the victim as the first step?

① Give water to the victim. ② Move the victim to a well-ventilated area.

③ Call 120 and send the victim to hospital. ④ I do not know.

**B07.** For the treatment of tuberculosis patients, which one of the following statements is correct:

① No special policy. ② Anti-tuberculosis drugs are provided by the governments for free.

③ Treatment for uberculosis are free at hospitals. ④ I do not know.

**B08.** When engaging in toxic and hazardous operations, one should:

① Wear work clothes. ② Wear safety helmet.

③ Use personal occupational disease protection equipment. ④ I do not know.

**B9.** The main hazard of iodine deficiency is:

① Suffering from “SARS”. ② Affect intelligence and body growth.

③ Cause high blood pressure. ④ I do not know.

**B10.** During intensive physical activities, body will experience excessive loss of the water through excessive sweating. In this case, it is best to take:

① Cooled boiled water. ② Sugar-sweetened beverages. ③ Light salt water. ④ I do not know.

**B11.** Regarding the National Basic Public Health Services, which of the following statements is incorrect:

① It is conducted in large hospitals.

② In urban areas, it is conducted in community healthcare centers (posts), and in rural areas, it is conducted in township healthcare centers (village clinics).

③ Every resident has free access. ④ I do not know.

**B12.** In which one of cases the vaccine should be suspended for children:

① Crying. ② Having a cold/fever. ③ Less than half an hour after the meal. ④ I do not know.

**B13.** When you have fever symptoms, the correct approach is:

① Go to the doctor promptly. ② Taking antipyretics based on your experience.

③ Observation before doing anything. ④ I do not know.

**B14.** When the patient has adverse reactions after taking the medicine following the doctor’s treatment plan, the correct approach is:

① Stop taking the medicine without consulting doctors. ② Consulting doctors.

③ Continue to take the medicine. ④ I do not know.

**B15.** A potent infectious disease broke out in an area. Which one of the following is correct:

① This disease has nothing to do with me and do nothing.

② If I were a local of that area, I would pay attention to the epidemic.

③ Whether or not I am a local, I need to pay attention to the epidemic.

④ I do not know.

**B16.** The warning sign
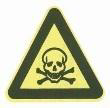
means:

① The site is prone to fire.

② There are explosives near the site, and one should avoid getting close to the site.

③ The article with the sign is toxic or there are toxic substances on the site.

④ I do not know.

**B17.** The national toll-free health hotline is:

① 12315. ② 120. ③ 12320. ④ I do not know.

**B18.** About seeking medical treatment, which one of the following statement is incorrect:

① Explain fully to your doctor about your conditions.

② If you have previous medical records, examination results, etc., it is best to bring them with you when you go to the doctor.

③ In order to get the attention of the doctor, you can describe your condition more serious than it actually is.

④ I do not know.

**B19.** A drug with the “OTC” logo printed on the package is:

① Prescription drugs, which must be prescribed by a doctor.

② Over-the-counter drugs, which you can buy without a doctor’s prescription.

③ Healthcare products. ④ I do not know.

**B20.** During the flu seasons, rooms should be ventilated regularly by opening windows. Which one of the following statements is incorrect about ventilation?

① In winters windows should be closed as much as possible to avoid catching colds.

② Ventilation by opening windows can dilute indoor bacteria and viruses.

③ Opening windows can allow sunlight to get into the room, which kills a variety of bacteria and viruses.

④ I do not know.

**B21.** When measuring body temperature using a mercury thermometer, the correct reading method is:

① Read by holding horizontally the mercury end of the thermometer.

② Read by holding vertically the glass end of the thermometer.

③ Read by holding horizontally the glass end of the thermometer. ④ I do not know.

**B22.** When the skin is mildly scalded, and blisters appear, which one of the following is correct:

① Pop the blisters, so you can recover faster.

② Small blisters do not need to be popped, but large blisters should be popped.

③ Do not pop the blisters to avoid infection. ④ I do not know.

**B23.** In the event of a fire, which one of the following responses is correct:

① Encase your head with your arms or clothes and rush out of the fire.

② You should not escape by using the elevator.

③ Evacuate while fighting the flames with clothes.

④ I do not know.

**3. Multiple choice questions with more than one answer. (each question has 2 or more correct options, please tick “√” on the corresponding option numbers. If you do not know, select 5).**

**C01.** Regarding ways to promote mental health, which of the following are true:

① Take an optimistic attitude towards one’s life.

② Set attainable goals.

③ Establish good interpersonal relationships and actively participate in social activities.

④ Relieve anxiety by smoking and drinking.

⑤ I do not know.

**C02.** Which of the following statements are correct about seeking medical treatment:

① Not all diseases can be cured. ② It’s the doctor’s job to treat the disease, not the patient’s.

③ Hospitals are the place to treat the disease, and it is the responsibility of hospitals to cure the diseases.

④ Birth, ageing, sickness and death are parts of the nature and one should treat the results of diagnoses and treatments rationally. ⑤ I do not know.

**C03.** Regarding the liver, which of the following are true:

① Can secrete bile. ② Has detoxification function.

③ It is an important digestive organ of the human body.

④ There are two livers (left and right). ⑤ I do not know

**C04.** If the child has symptoms such as fever and rash, parents should:

① Go to the hospital promptly. ② Stop going to the kindergarten.

③ Notify the teacher in the kindergarten promptly. ④ Send the child to the kindergarten as usual. ⑤ I do not know.

**C05.** When purchasing packaged food, what information on the package should one pay attention to?

① Production date. ② Expiration date. ③ Nutrition table.

④ Manufacturer. ⑤ I do not know.

**C06.** If sick or dead livestock are found, the following should be done:

① No slaughter, no processing. ② No sale, and no transportation. ③ Do not eat.

④ Can be eaten after being well cooked. ⑤ I do not know.

**C07.** What measures should be taken when you suspect someone experiences respiratory or cardiac arrest?

① Artificial respiration. ② Chest compressions. ③ Make an emergency call.

④ Administer high blood pressure medication. ⑤ I do not know.

**C08.** The benefits of eating soybean products such as tofu and soymilk are:

① It is good for your health.

② It is good for people with cardiovascular diseases.

③ It is source of good proteins.

④ It can be used as treatment for certain diseases. ⑤ I do not know.

**C09.** Health benefits of exercise include:

① Weight control. ② Prevention of chronic diseases. ③ Reduce stress.

④ Improve quality of sleep. ⑤ I do not know.

**C10.** A newspaper reads that any diabetic patient can be completely cured by taking an oral hypoglycemic product. After reading this message, which of the following descriptions are correct?

① This news is not credible.

② This is good news. Hurry up and tell your diabetic friends.

③ Consult and verify with the community doctor.

④ Buy without hesitation. ⑤ I do not know.

**C11.** When coughing or sneezing, the correct way to do this are:

① Cover your mouth and nose directly with your hands. ② Cover your mouth and nose with a handkerchief or a paper towel.

③ Cover your mouth and nose with your elbows. ④ Do not cover your mouth and nose.

⑤ I do not know.

**C12.** Which of the following statements are correct about seeking medical treatment:

① When you are sick, you should go to the hospital to see doctors.

② Your first choice is to go to the community healthcare centers for diagnosis and treatment. Do not go to the hospital unless it is necessary.

③ You should return to the community health centers in the stage of rehabilitation for disease management.

④ You should go to the hospital in the stage of rehabilitation. ⑤ I do not know.

**C13.** What are benefits of breastfeeding?

① Breastfeeding can make babies less sick. ② Breast milk is the best natural food for babies.

③ Infant formula is more nutritious than breast milk.

④ Breastfeeding can improve the mother-baby bonding and enhance emotional development in the baby. ⑤ I do not know.

**C14.** When storing pesticides, caution that:

① Pesticide should be kept in the safe and appropriate place.

② Pesticide cannot be placed together with food.

③ If your hands are contaminated with pesticides, do not wash if the skin is not broken.

④ Pesticide should be kept out of the reach of children. ⑤ I do not know

**C15.** When there is lightning and you are outdoors, which of the following are correct:

① Shelter under a tree. ② Stay away from power lines. ③ Avoid using cell phones.

④ Go to elevated areas. ⑤ I do not know.

**4. Vignette Questions. (please read the following passage and answer the questions)**

BMI refers to the body mass index, which is commonly used internationally as a standard to measure the degree of fitness of the human body. The calculation of BMI is dividing weight in kilograms by height in meter squared, that is, BMI = weight / height^2^ (kg/m^2^). For Chinese adults, BMI < 18.5 is considered underweight, 18.5 ≤ BMI < 24 is normal, 24 ≤ BMI < 28 is overweight, and BMI ≥ 28 is obese.

**D01.** Mr. Li is 45 years old with a height of 170 cm and weighs 80 kg. How to calculate his BMI? (single-answer)

① (80)^2^ / 170 = 37.6. ② 80 / (1.7)^2^ = 27.2. ③ 160 / (170)^2^ = 55.4. ④ I do not know.

**D02.** Considering the BMI categorization for Chinese adults, Mr. Li is categorized as: (single-answer)

① Obesity. ② Normal weight. ③ Overweight. ④ I do not know.

**D03.** To help Mr. Li control weight, which of the following methods can be used? (multiple-answer)

① Do not eat rice. ② Daily exercise for at least 30 min. ③ Reduce fat intake.

④ Eat only vegetables and fruits. ⑤ I do not know.

**D04.** Which of the following diseases might Mr. Li get? (single-answer)

① Hypertension. ② Osteoporosis. ③ gastric ulcer. ④ I do not know.
